# Supplementary material for: Resemblance of the Recurrence Patterns in Primary Systemic, Primary Surgery and Secondary Oncoplastic Surgery
Source: Curr Oncol. 2022 Nov 17;29(11):8874–85. doi: 10.3390/curroncol29110698 (PMC9689416; doi:10.3390/curroncol29110698)
Supplement: Supplementary file 1 [file curroncol-29-00698-s001.zip › curroncol-2025042-supplementary.pdf]

General analysis of the groups

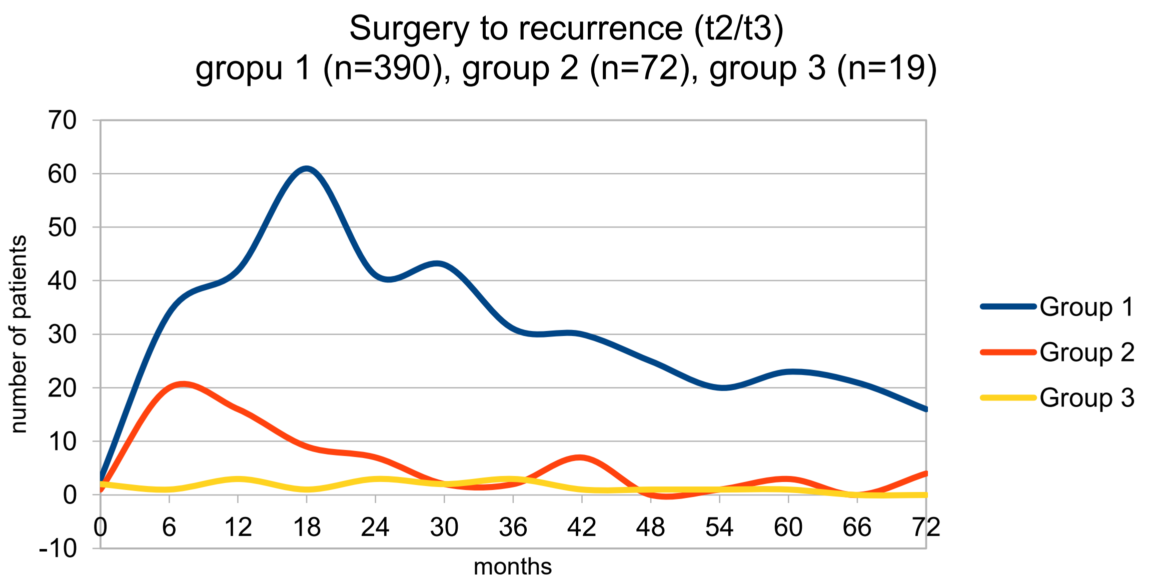

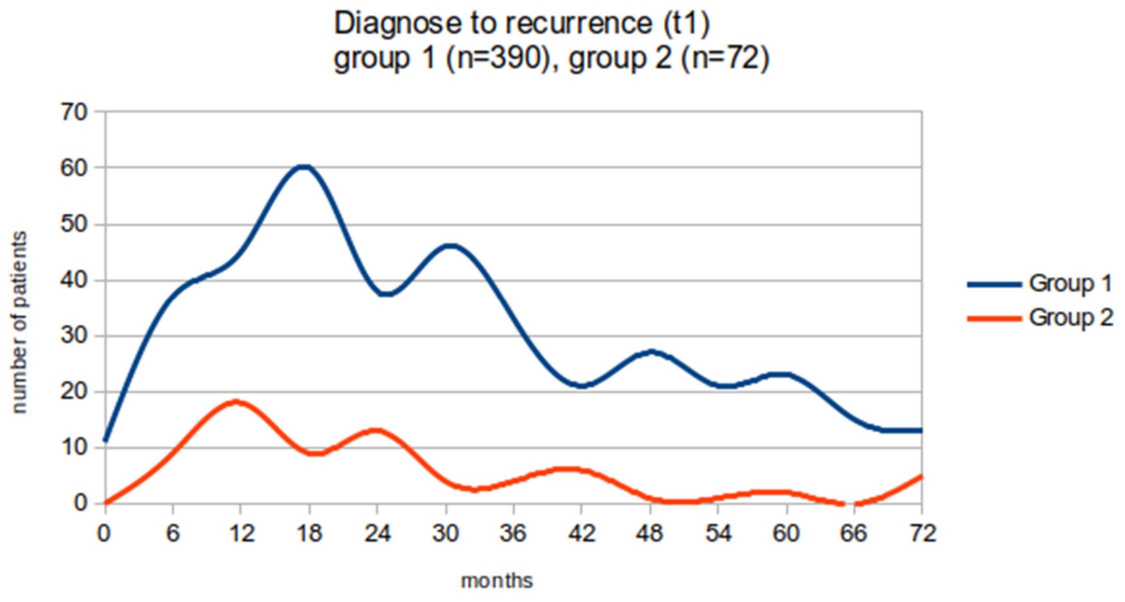

General analysis of group 1

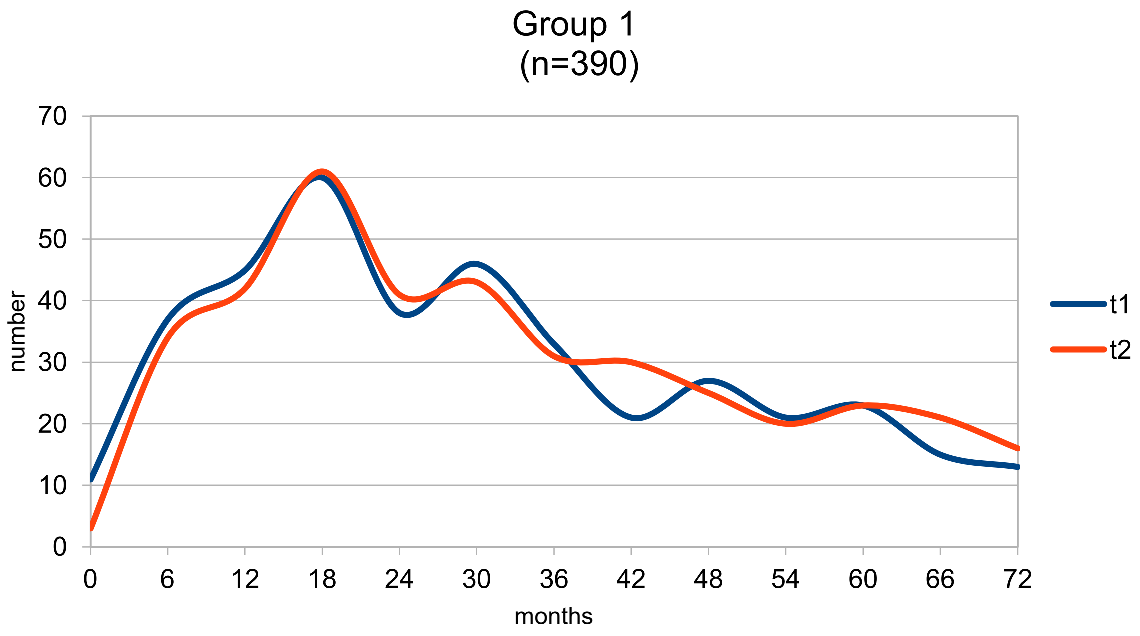

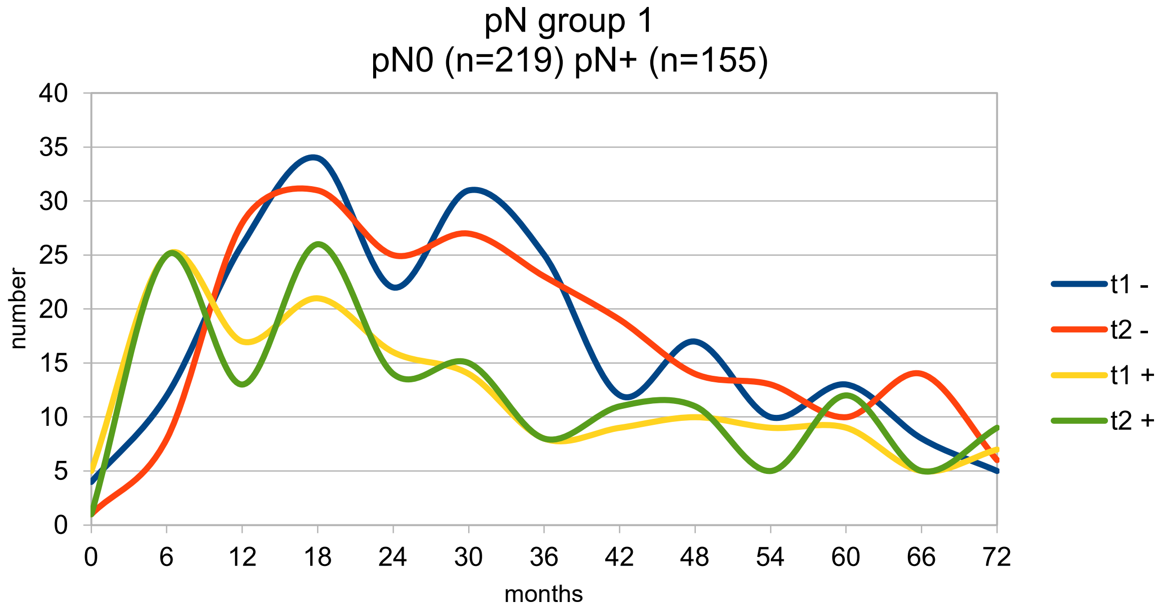

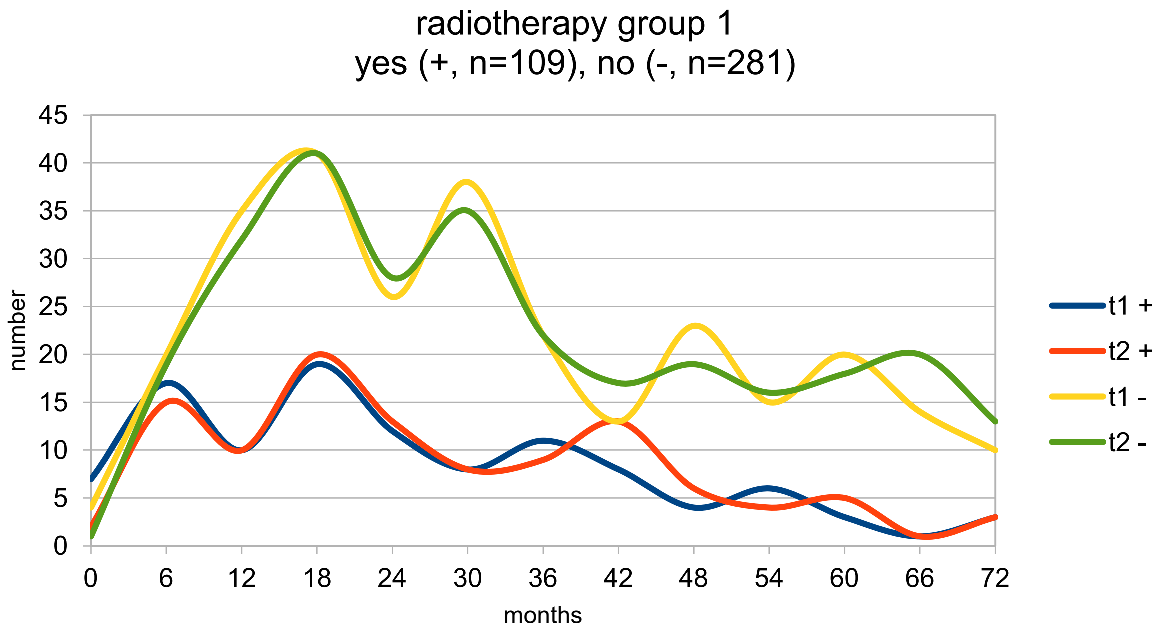

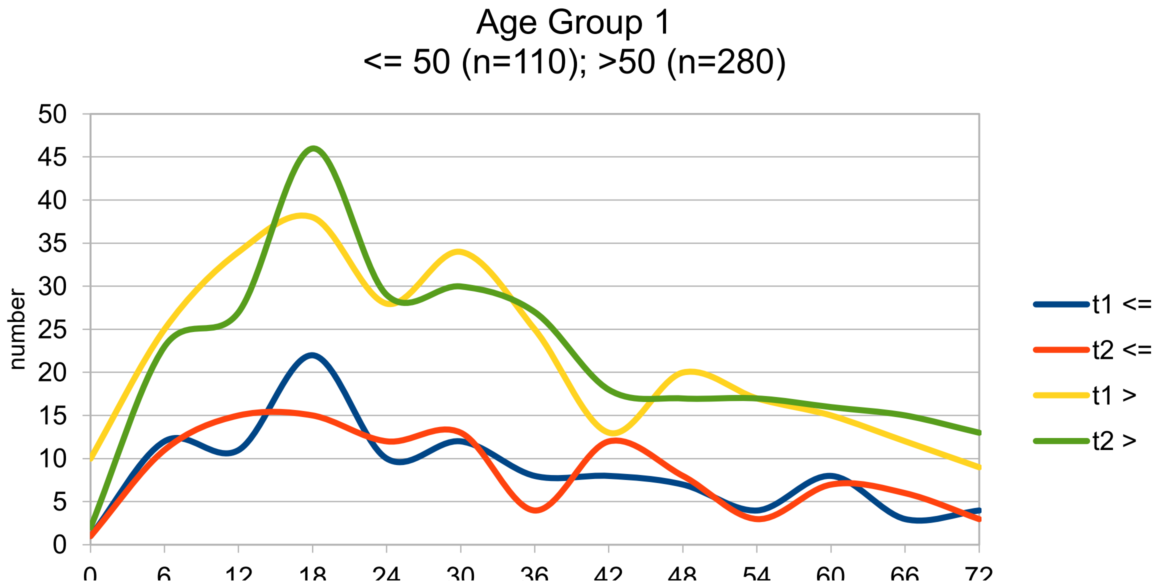

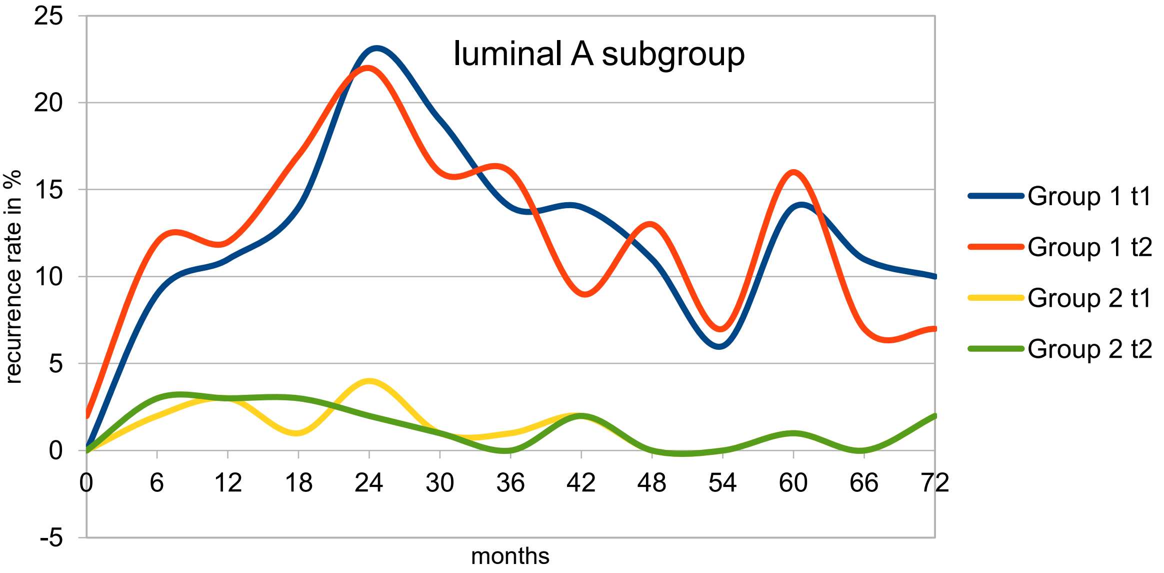

Subg
roup
analy
sis of
the
recur
rence
rates

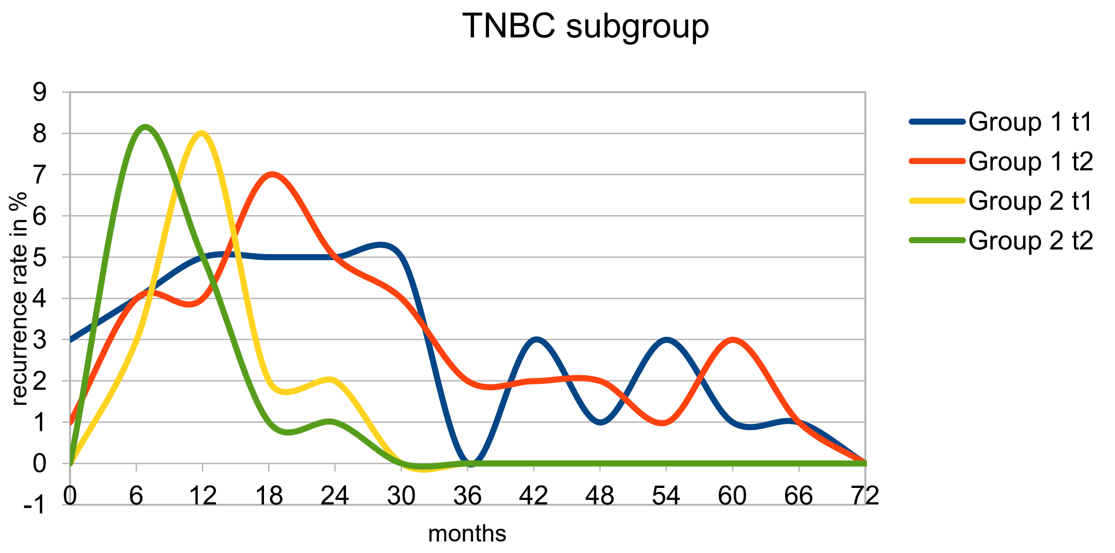
TNBC subgroup
recurrence rate in %
months
Group 1 t1
Group 1 t2
Group 2 t1
Group 2 t2

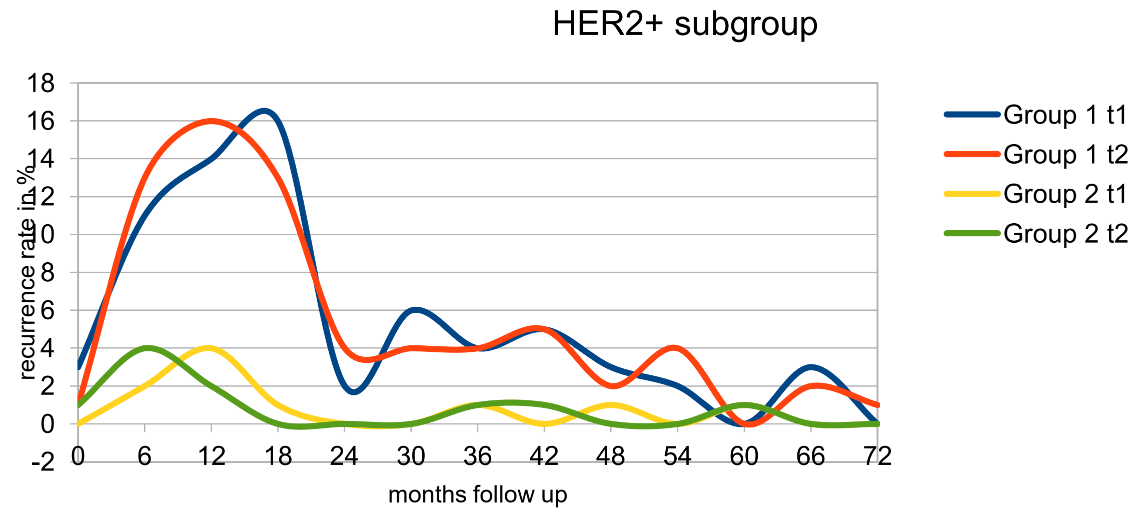
HER2+ subgroup
recurrence rate in %
months follow up
Group 1 t1
Group 1 t2
Group 2 t1
Group 2 t2

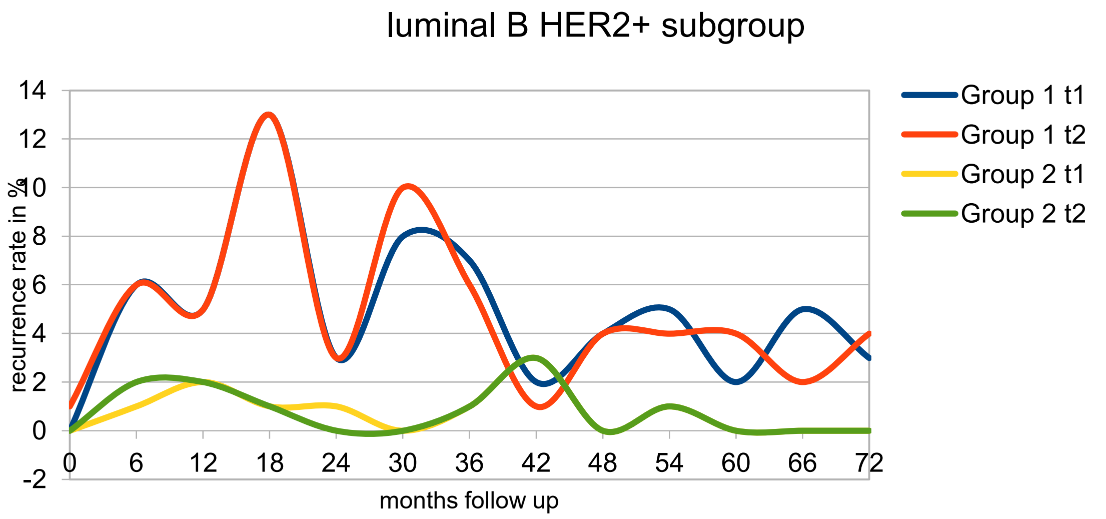
luminal B HER2+ subgroup
recurrence rate in %
months follow up
Group 1 t1
Group 1 t2
Group 2 t1
Group 2 t2

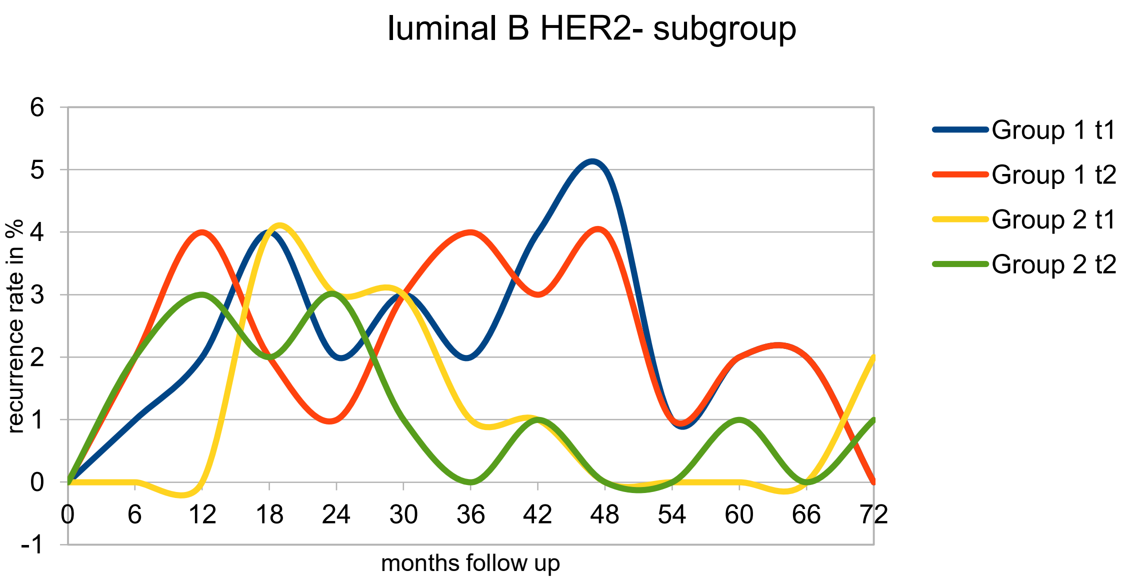

luminal B HER2- subgroup
6
5
4
3
2
1
0
-1
recurrence rate in %
0
6
12
18
24
30
36
42
48
54
60
66
72
months follow up
Group 1 t1
Group 1 t2
Group 2 t1
Group 2 t2

General analysis of group 2

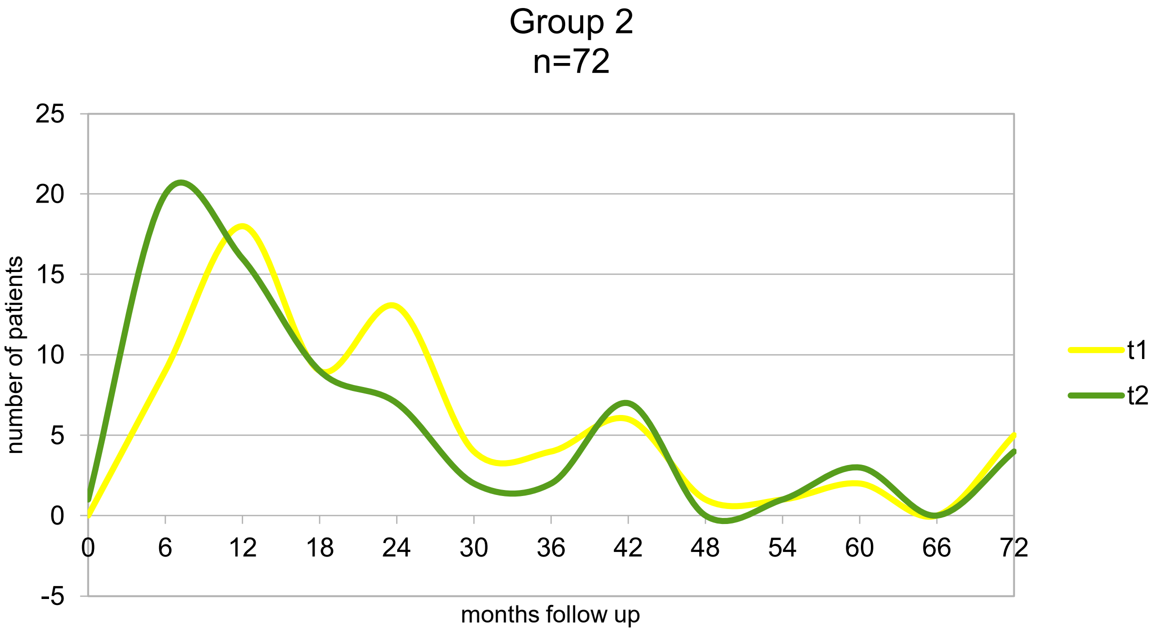

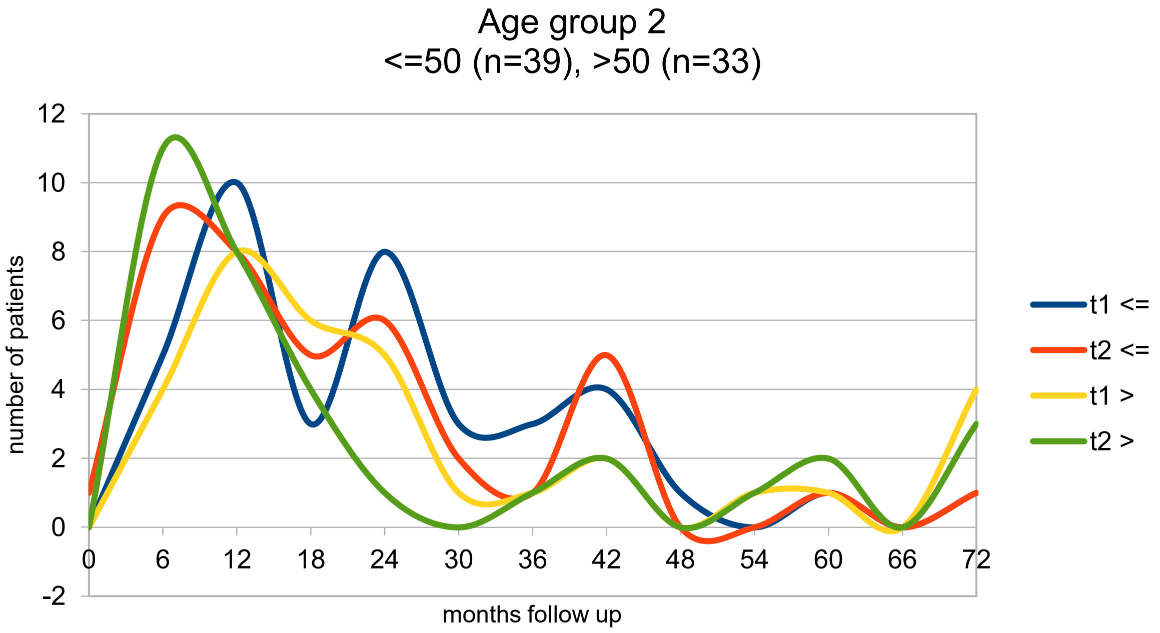

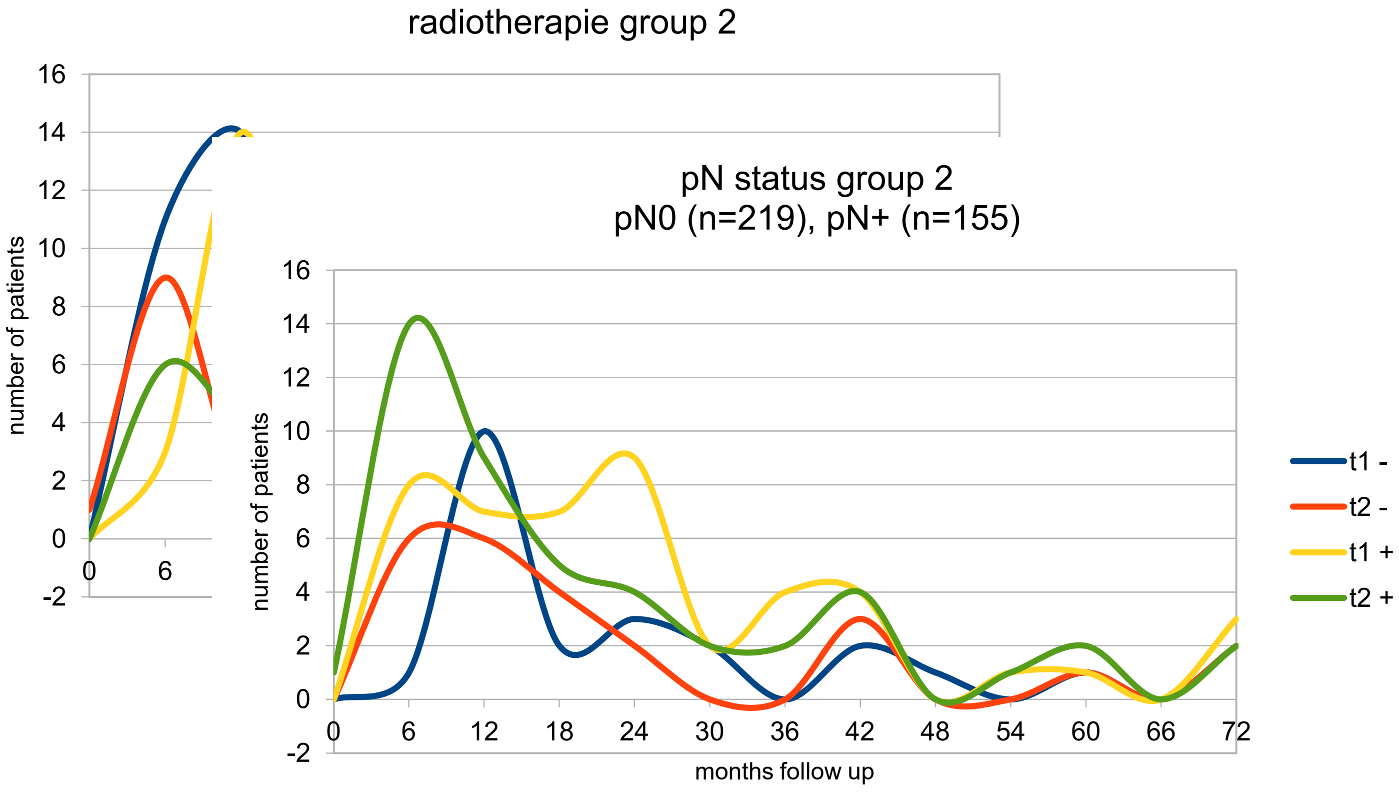
radiotherapie group 2
pN status group 2
pN0 (n=219), pN+ (n=155)
number of patients
months follow up
16
14
12
10
8
6
4
2
0
-2
0
6
12
18
24
30
36
42
48
54
60
66
72
t1 -
t2 -
t1 +
t2 +
